# Differential coupling of gibberellin responses by *Rht-B1c* suppressor alleles and *Rht-B1b* in wheat highlights a unique role for the DELLA N-terminus in dormancy

**DOI:** 10.1093/jxb/erw471

**Published:** 2017-01-09

**Authors:** Karel Van De Velde, Peter Michael Chandler, Dominique Van Der Straeten, Antje Rohde

**Affiliations:** 1 R&D Innovation Center, Bayer CropScience, Ghent, Belgium; 2 Ghent University, Department of Physiology, Laboratory of Functional Plant Biology, Ghent, Belgium; 3 CSIRO Plant Industry, Canberra, ACT, Australia

**Keywords:** DELLA, dormancy, gibberellin (GA), pre-harvest sprouting, suppressor alleles, wheat

## Abstract

During the Green Revolution, substantial increases in wheat (*Triticum aestivum*) yields were realized, at least in part, through the introduction of the Reduced height (*Rht*)*-B1b* and *Rht-D1b* semi-dwarfing alleles. In contrast to *Rht-B1b* and *Rht-D1b*, the *Rht-B1c* allele is characterized by extreme dwarfism and exceptionally strong dormancy. Recently, 35 intragenic *Rht-B1c* suppressor alleles were created in the spring wheat cultivar Maringá, and termed *overgrowth* (*ovg*) alleles. Here, 14 *ovg* alleles with agronomically relevant plant heights were reproducibly classified into nine tall and five semi-dwarf alleles. These alleles differentially affected grain dormancy, internode elongation rate, and coleoptile and leaf lengths. The stability of these *ovg* effects was demonstrated for three *ovg* alleles in different genetic backgrounds and environments. Importantly, two semi-dwarf *ovg* alleles increased dormancy, which correlated with improved pre-harvest sprouting (PHS) resistance. Since no negative effects on grain yield or quality were observed, these semi-dwarf *ovg* alleles are valuable for breeding to achieve adequate height reduction and protection of grain quality in regions prone to PHS. Furthermore, this research highlights a unique role for the first 70 amino acids of the DELLA protein, encoded by the *Rht-1* genes, in grain dormancy.

## Teaser

Characterization of *Rht-B1c* suppressor alleles in different cultivars and environments highlights a unique role of the N-terminal domain of DELLA in grain dormancy, and reveals opportunities for improving pre-harvest sprouting resistance.

## Introduction

Mutations in key regulators of the gibberellin (GA) pathway formed the genetic basis of a global agricultural revolution in the mid and late 20th century. During the so-called ‘Green Revolution’, yields of rice and wheat greatly increased due to intensified use of fertilizers and pesticides, combined with the introduction of semi-dwarfing alleles (Khush, 1999; Hedden, 2003). These alleles reduced plant height, which allowed higher fertilizer rates due to improved lodging resistance, and resulted in increased grain number per spike (Youssefian *et al.*, 1992). In wheat, the Green Revolution semi-dwarfing alleles derive from a *Della* gene, encoded by the *Reduced height* (*Rht-1*) locus (Peng *et al.*, 1999). In hexaploid bread wheat, *Della* is encoded by three homoeologous genes (*Rht-A1*, *Rht-B1*, and *Rht-D1*), with the wild-type alleles designated *Rht-A1a*, *Rht-B1a*, and *Rht-D1a*, respectively. The Green Revolution semi-dwarfing alleles of the B- and D-subgenome, *Rht-B1b* and *Rht-D1b* (previously named *Rht1* and *Rht2*, respectively), are now found in wheat cultivars grown worldwide. Both contain a mutation in the region of the DELLA protein’s eponymous N-terminal DELLA motif (Peng *et al.*, 1999). This motif is crucial for degradation of the DELLA protein, which is triggered upon the detection of bioactive GAs by the GA receptor, GIBBERELLIN INSENSITIVE DWARF1 (GID1) (Davière and Achard, 2013). Although *in planta* biochemical evidence is still missing, yeast two-hybrid analysis has shown that the interaction between the DELLA protein and GID1 is reduced in the *Rht-B1b* mutant, presumably resulting in an accumulation of the DELLA protein (Pearce *et al.*, 2011; Wu *et al.*, 2011).

Besides elongation, DELLA controls the synthesis of hydrolytic enzymes in the grain aleurone layer (Richards *et al.*, 2001). Hydrolytic enzymes, such as α-amylase, can occasionally be present in the mature grain, and affect the bread-making quality of wheat flour. Excessive α-amylase will degrade starch during the baking process, leading to a sticky and difficult to raise dough, and discolored, poorly structured loaves (Edwards *et al.*, 1989). In practice, α-amylase activity is often indirectly assessed by the falling number, which is the time it takes an object to fall through an aqueous slurry of flour. The viscosity of the flour solution depends on the amount of long-chain carbohydrates. The lower the viscosity, which mainly results from the breakdown of long-chain carbohydrates by α-amylase activity, the lower the falling number (Perten, 1964). Elevated levels of α-amylase in the mature grain can originate from pre-harvest sprouting (PHS), which occurs when wet conditions prior to harvest break dormancy and induce grain germination while in the ear (Trethowan, 2001). Although PHS results in worldwide losses of more than US$1 billion per year (Black *et al.*, 2006), the strong interaction between PHS and environmental and genetic factors often hampers selection for PHS resistance in conventional breeding programs (Trethowan, 2001; Groos *et al.*, 2002; Mares and Mrva, 2014). Therefore, it is mandatory to study PHS resistance in different environments and genetic backgrounds.

The *Rht-B1c* allele contains a 2 kb insertion which, after splicing, results in an in-frame insertion of 30 amino acids, adjacent to the DELLA motif. This insertion completely disrupts the interaction between the RHT-B1C protein and GID1, resulting in GA insensitivity (Pearce *et al.*, 2011; Wu *et al.*, 2011). In contrast to *Rht-B1b* and *Rht-D1b*, the *Rht-B1c* allele is characterized by exceptionally strong dormancy and extreme dwarfism, the latter preventing its use in commercial cultivars (Flintham and Gale, 1982; Flintham *et al.*, 1997*b*). However, a screen for second-site suppressor mutants in the cultivar Maringá identified 35 new *Rht-B1c* derivative alleles (Chandler and Harding, 2013, 2014). These alleles were termed *overgrowth* (*ovg*) alleles and contained, besides the 30 amino acid insertion, second-site mutations in other regions of the gene. The *ovg* alleles partially restore primary GA responses, such as stem length, coleoptile length, and dormancy.

Here, we assess the GA-related effects in different plant organs for 14 Maringá *ovg* alleles that result in agronomically relevant plant heights. To validate further the observed *ovg* effects and to evaluate the breeding potential of selected alleles, a subset of *ovg* alleles was introgressed into four spring wheat cultivars. Phenotyping in the greenhouse and field demonstrated the consistency of the *ovg* effects and, importantly, revealed no negative effects on grain yield or quality. In addition, *Rht-B1c.23* and *Rht-B1c.26* showed increased dormancy and improved PHS resistance. We conclude that these *ovg* alleles are valuable for breeding to achieve adequate height reduction and protection of grain quality in regions prone to PHS. Furthermore, the phenotypic effect of specific amino acid residues that are mutated in the *ovg* alleles aids our understanding of the regulation of GA responses by the DELLA protein in different plant organs. In addition, a unique role of DELLA’s first 70 amino acids in grain dormancy is highlighted.

## Materials and methods

### Plant material

In the Brazilian bread wheat cultivar Maringá, sequencing of the *Rht-B1* gene confirmed the genotype of tall (*Rht-B1a*), semi-dwarf (*Rht-B1b*), and dwarf (*Rht-B1c*) backcross (BC) 7 isolines, and the second-site mutations in the 14 *ovg* mutants (see Supplementary Table S1 at *JXB* online; Chandler and Harding, 2013, 2014).

Maringá *ovg* mutants containing *Rht-B1c.22*, *Rht-B1c.23*, and *Rht-B1c.26* were backcrossed to the spring wheat cultivars KWS Scirocco and Faller, derived from Europe and North America, respectively. While both cultivars carry the wild-type *Rht-D1a* allele, KWS Scirocco contains wild-type *Rht-B1a*, whereas Faller carries the *Rht-B1b* semi-dwarf allele. BCF_1_ plants were allowed to self when at least 90% of the 384 single nucleotide polymorphisms (SNPs), tested via Kompetitive Allele Specific PCR (KASP), were inherited from the recurrent parent. To this end, four backcrossing generations were required, except for *Rht-B1c.23* and *Rht-B1c.26* in KWS Scirocco, which required only three backcrossing generations. KASP designed against *Rht-B1c*-specific SNPs (Pearce *et al.*, 2011) allowed selection of sister lines of four different BCF_2_ families, homozygous for either the *ovg* or the *Rht-B1b/Rht-B1a* allele. Phenotypes were assessed at the F_3_ generation in the greenhouse and the F_4_ generation in the field.

Maringá *ovg* mutants containing *Rht-B1c.23* and *Rht-B1c.26* were backcrossed to the Australian spring wheat cultivars Crusader and EGA Gregory, both of which carry the *Rht-D1a* wild-type and *Rht-B1b* semi-dwarfing allele. Seedlings of at least three different BC2F_2_ families were genotyped to identify those homozygous for either the *ovg* or the *Rht-B1b* allele, and they were selfed until BC2F_5_ grains were obtained. Since Maringá has red grains, while Crusader and EGA Gregory have white grains, grain color segregated in this BC population. Therefore, the BC2F_5_ grains were scored for grain color after a 30 min treatment with 0.1 M KOH. Only white-grained sister lines, showing <2 d difference in anthesis compared with the recurrent parent, were selected for further study.

### Plant growth conditions

After 2–6 d of stratification at 4 °C, Maringá or BCF_3_ Faller/KWS Scirocco grains were sown in 4 liter pots filled with a perlite-containing compost-based mix (DCM^®^). The pots were white to reduce excessive heating of soil and roots by irradiation (Passioura, 2006). Plants were grown on the conveyor belts of a LemnaTec 3D-Scanalyzer in a greenhouse (21–19 °C) with incident day light and day length extension to a 16 h photoperiod when necessary (400 W high-pressure sodium lamps, GE Lucalox™). During the first week, rain water was applied daily, while for the remainder of the experiment the plants were watered daily with fertilizer solution up to a pre-defined individual pot target weight. This target weight was calculated as a percentage of soil water content at retention capacity (SRC), as described in Granier *et al.* (2006). After flowering, plants were transferred to a standard greenhouse compartment and watered daily with fertilizer solution via an intermittent subirrigation system (commercially referred to as an ebb-and-flow system). All experiments were conducted with at least 10 biological replicates in a randomized block design, without altering the position of the plants during the experiment, as recommended by Brien *et al.* (2013).

Field experiments involving KWS Scirocco and Faller (BCF_4_) were run in Gatersleben (Germany). Plots of 1.54 m×4 m were sown on 4 April 2015 in a randomized block design consisting of three replicated plots for each sister line. Phenotypic measurements were executed on six plants per plot; however, yield was determined per plot.

Field trials involving Crusader and EGA Gregory were executed in New South Wales, Australia. Grain multiplication and dormancy studies were carried out with BC2F_6_ grains from a field nursery at Canberra in 2014. In Yanco, BC2F_7_ plots of 1.62 m×5 m were sown on 4 June 2015, and were irrigated until grain development.

### Phenotypic measurements

To quantify coleoptile length, grain stratification at 4 °C for 2 d was followed by germination in the dark in soil at a constant temperature of 20 °C. When 50% of the first or the second leaf was visible in Maringá or backcrossed lines (Faller and KWS Scirocco), respectively, etiolated coleoptile length was measured. Zadoks phenology stages were determined as described in Zadoks *et al.* (1974), and the first leaves were measured from the ligule to the leaf tip. Furthermore, the main stem was marked at spike emergence (Zadoks 50) and its length was measured after flowering, from the soil level to below the spike, whereas peduncle length was determined from above the highest node to below the spike. At flowering (Zadoks 65), the lengths of the flag leaf sheath and flag leaf lamina were measured from above the highest node up to the ligule and from the ligule to the flag leaf tip, respectively. Maximal flag leaf width was measured at the basal part of the leaf. Total plant height during growth was calculated as the average plant height of two side view images taken at a 90 ° horizontal rotation (Chen *et al.*, 2014; Neumann *et al.*, 2015). Mature spikes of individual plants were cut, dried for at least 3 d at 30 °C, and threshed (SRC SAS, Mayet, France). The organ length of the main stem generally correlated with the organ length of tiller 1 and 2 (Supplementary Table S2). Subsequently, all grains were counted (Contador, Pfeuffer, Kitzingen, Germany), weighed, and the thousand grain weight (TGW) of individual greenhouse-grown plants or field plots was calculated. Grain length and width of at least 150 grains per line were determined with VideometerLab 3 (Hørsholm, Denmark), as described in Hansen *et al.* (2015).

### Dormancy

Physiologically mature spikes were harvested and dried for several days at 30 °C. Then, grains were hand threshed and stored at 30 °C. Hand threshing was required because machine threshing broke down dormancy completely. Each week, 100 grains were distributed on moist germination paper (AllPaper T10D li blue, 550 g m^−2^) in square Petri dishes (245 mm×245 mm, Sigma-Aldrich^®^). After 7 d of incubation at 20 °C under constant fluorescent light (50 µmol m^−2^ s^−1^), grain germination was assessed by radicle protrusion, and the germination percentage was calculated. Machine-threshed wild-type grains stored at 10 °C for >6 months were included as positive controls. To improve statistical power, the dormancy test of back-crossed Faller and KWS Scirocco lines was executed in a randomized block design by sorting 96 grains on three round Petri dishes (15 cm diameter, Falcon^®^) filled with agar medium containing 0.2% GELRITE™ G1101 (Duchefa Biochemie BV, Haarlem, The Netherlands), supplemented with 0.94 g l^–1^ MgCl_2_·6H_2_O to improve coagulation. Prior to germination, grains were sterilized with 3% NaOCl_2_.

### Pre-harvest sprouting

Spikes were harvested during grain ripening, dried at 30 °C, and positioned upright in a growth chamber (16 h day length, 22/18 °C day/night temperature, 70 µmol m^−2^ s^−1^). Every 1.5 h or 2 h during day or night, respectively, nozzles simulated strong rainfall for 10–15 s. When spikes sprouted, pictures were taken.

### Falling number

Grain samples were stored until germination tests indicated that dormancy was completely lost (i.e. grains germinated >95%). Subsequently, the falling number of 100 g of grains per replicate field plot or 30 g of bulked, greenhouse-harvested grains (split into three 10 g samples) was determined according to the international standard method, ICC-No. 107/1.

### Data analysis

Statistics were performed with R (R Development Core Team, 2008). Using a mixed model, the effect between the *ovg* alleles and the reference allele (*Rht-B1a* or *Rht-B1b*) was estimated, adjusting for irrigation treatment and block. The analysis model was also used to remove potential outliers that have a Studentized residual value >2. All effects with *P*<0.05 were considered significant.

## Results

### Effect of *ovg* alleles on elongation

From the 35 Maringá *ovg* alleles (Chandler and Harding, 2013, 2014), 14 *ovg* alleles that resulted in maximal 40% height reduction, relative to *Rht-B1a*, were selected as agronomically relevant. To compare these 14 *ovg* alleles with the widely deployed *Rht-B1b* allele, the final length of plant organs in different cultivars and environments was studied, and expressed relative to *Rht-B1b* (Table 1). In greenhouse-grown Maringá plants, the stem lengths of nine *ovg* alleles were 12–34% longer than those of *Rht-B1b*, while the stem lengths of five other *ovg* alleles were either ~10–15% shorter than, or not significantly different from *Rht-B1b* (Table 1). These *ovg* alleles were classified as tall and semi-dwarf alleles, respectively. Similar to their effect on stem length, tall and semi-dwarf alleles proportionally affected the final length of the coleoptile and peduncle (Table 1).

**Table 1.**
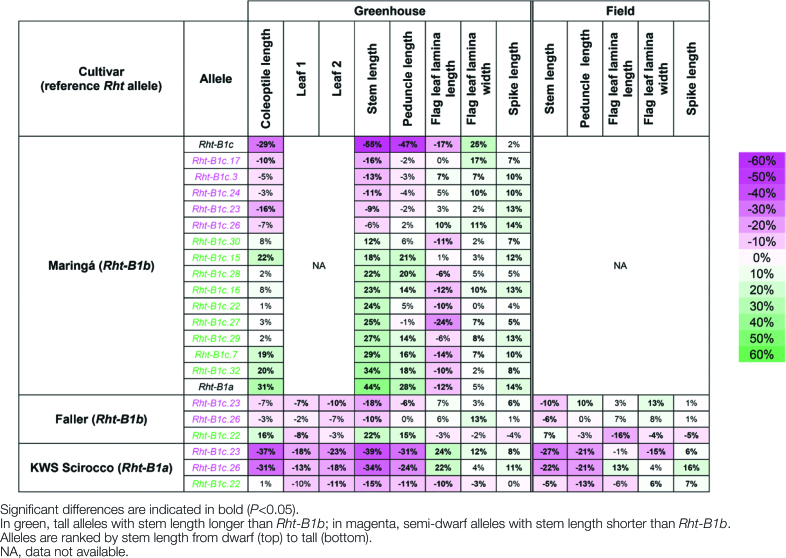
Effect of *ovg* alleles on elongation in different wheat cultivars, in the greenhouse and field Percentage average difference of phenotypic traits for each *ovg* allele, relative to the reference allele, are shown. The scale next to the table details the intensity of color saturation for percentage difference.

**Fig. 1.**
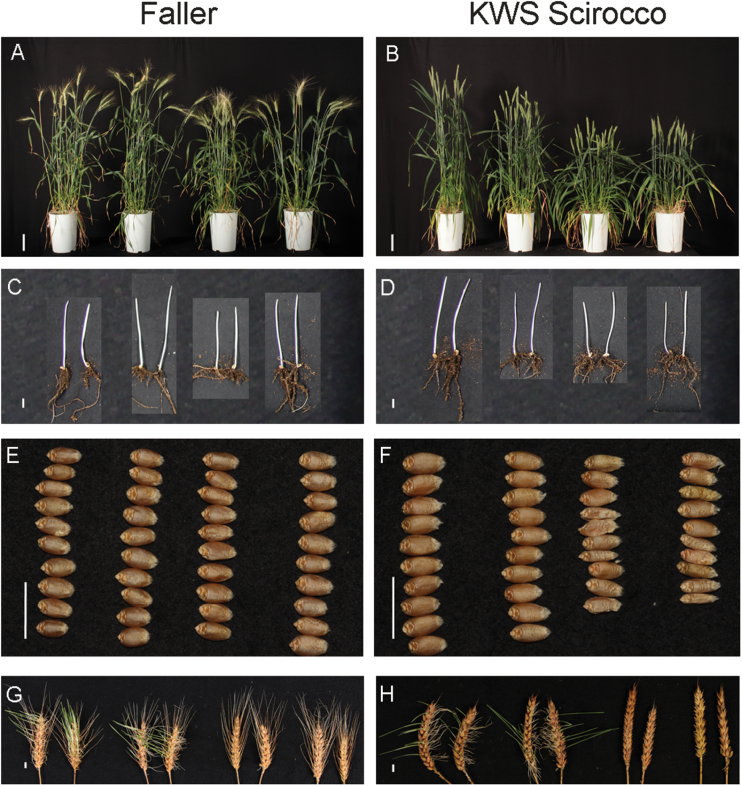
Phenotypic effects of three *ovg* alleles, compared with the respective reference allele, in Faller and KWS Scirocco. From left to right: reference allele (*Rht-B1b* for Faller, *Rht-B1a* for KWS Scirocco), *Rht-B1c.22*, *Rht-B1c.23*, and *Rht-B1c.26*. (A, B) Flowering plants 3 months after sowing; scale bar=10 cm. (C, D) Etiolated coleoptiles when 50% of the second leaf was visible; all leaves were removed on the picture; scale bar=1 cm. (E, F) Ten grains; scale bar=1 cm. (G, H) Sprouted spikes, harvested at physiological maturity from plants grown in the greenhouse; scale bar=1 cm.

To validate the *ovg* effects in other genetic backgrounds, one tall (*Rht-B1c.22*) and two semi-dwarf (*Rht-B1c.23* and *Rht-B1c.26*) alleles were introgressed into the spring wheat cultivars Faller and KWS Scirocco, developed for North America and Europe, respectively. KWS Scirocco contains the wild-type *Rht-B1a* allele at the *Rht-B1* locus, whereas Faller contains the *Rht-B1b* allele. In both cultivars, homozygous sister lines carrying either the *ovg* or the wild-type (*Rht-B1a* or *Rht-B1b*, respectively) allele were generated from at least three BCF_1_ plants to assess the *ovg* effect rigorously. Herein, results of one sister line pair will be displayed, but all other pairs showed similar results. In greenhouse-grown Faller, the coleoptile, peduncle, and stem lengths of lines containing *Rht-B1c.22* were 15–22% longer than those of *Rht-B1b*, while for the lines containing *Rht-B1c.23* and *Rht-B1c.26*, these parameters were either up to 18% shorter than or not significantly different from *Rht-B1b* (Table 1; Fig. 1A, C). In KWS Scirocco, the length reduction of the coleoptile, stem, and peduncle was greater for semi-dwarfing alleles than for the tall allele: stem length reduction of *Rht-B1c.23* and *Rht-B1c.26* was 39% and 34%, respectively, while it was only 15% for *Rht-B1c.22* (Table 1; Fig. 1B, D). For both cultivars, the differential effect on stem length was confirmed in field experiments (Table 1). Together, the observations show that the *ovg* effect on the elongation of vegetative organs is robust across different genetic backgrounds and environments.

**Fig. 2.**
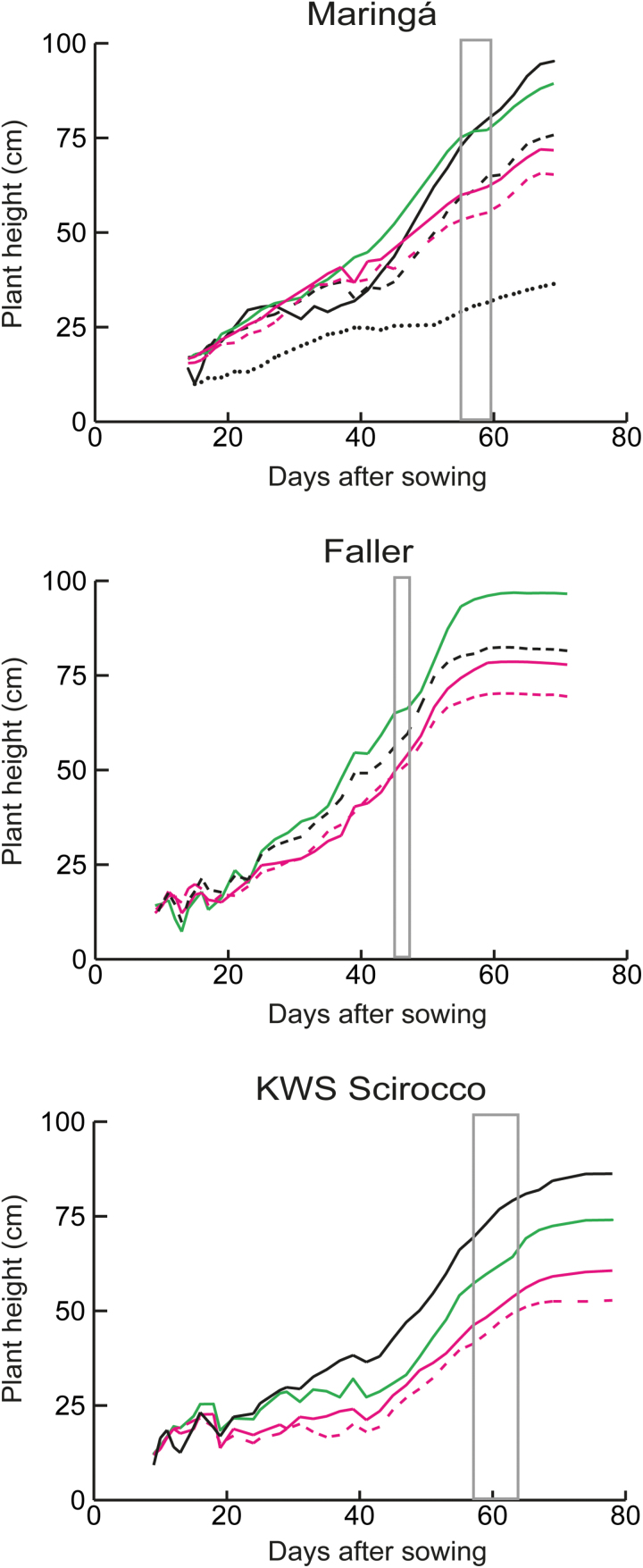
*ovg* alleles differentially affect plant elongation in different cultivars in the greenhouse. Plant heights are averages of at least 10 plants. The box indicates the period of first spike emergence (Zadoks 50). Black full line, *Rht-B1a*; black dashed line, *Rht-B1b*; black dotted line, *Rht-B1c*; full green line, *Rht-B1c.22*; full magenta line, *Rht-B1c.26*; dashed magenta line, *Rht-B1c.23*. Results were confirmed in two independent experiments. Representative data from the first experiment are shown.

To investigate further the effect of the *ovg* alleles, plant height and development were monitored on an automated phenotyping platform. In Maringá, *Rht-B1c.22* grew taller than *Rht-B1c.23* and *Rht-B1c.26* from 40 d after sowing—the onset of stem elongation—while these effects were visible 15 d earlier in Faller and KWS Scirocco (Fig. 2). These findings suggest that the *ovg* effect on final stem length results from an effect on each internode. Accordingly, the final length of each stem internode was proportionally affected by *Rht-B1c.26* in Maringá (Supplementary Fig. S1). Furthermore, the *ovg* alleles did not significantly affect timing of developmental events, indicated by the narrow window showing the onset of spike emergence (Fig. 2). Consequently, the *ovg* effect on final plant height resulted from a cumulative effect on internode elongation rate rather than from an effect on the total duration of elongation. In other words, internodes of semi-dwarf alleles always elongated more slowly than internodes of tall alleles. Interestingly, *Rht-B1c.23* elongated more slowly than *Rht-B1c.26* in all three cultivars, indicating subtle, reproducible differences between these two semi-dwarf alleles (Fig. 2).

### Effect of *ovg* alleles on yield-related traits

**Fig. 3.**
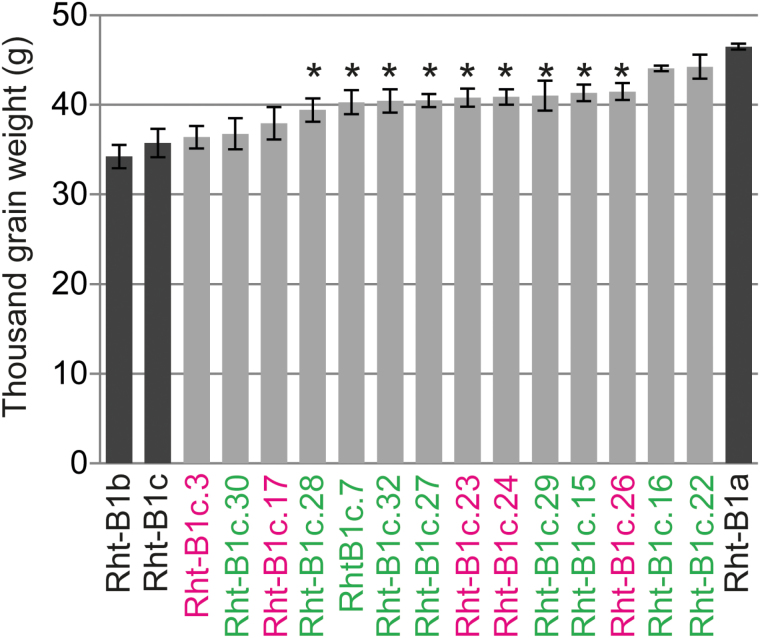
Effect of *ovg* alleles on TGW in greenhouse-grown Maringá. Shown are the average values for each allele ±SE (*n*=10). * indicates significant difference from *Rht-B1a* or *Rht-B1b* (*P*<0.05). Tall alleles are in green and semi-dwarf alleles are in magenta.

The robustness of the *ovg* effect in controlling plant height offers a potential for breeding applications: when the extent of dwarfing with the standard semi-dwarfing alleles is slightly too strong or too weak for a specific target environment, respectively, a tall or semi-dwarf *ovg* allele could be used instead. To determine whether there were any negative pleiotropic effects of the *ovg* alleles on yield-related traits, flag leaf dimensions, spike length, grain size, and grain yield were measured in the greenhouse and field (Tables 1, 2; Fig. 3). In Maringá, KWS Scirocco, and Faller, flag leaf lamina length was generally reduced by tall alleles, but increased by semi-dwarf alleles, with a maximum increase of 24% in greenhouse-grown KWS Scirocco (Table 1). This finding is opposite to the effect of the *ovg* alleles on the elongation growth of coleoptile and stem. In addition, the flag leaf width was generally increased by all *ovg* alleles, linking this effect to the primary *Rht-B1c* allele, which showed the strongest increase of 25%, thereby confirming previous studies (Wen *et al.*, 2013). Since the flag leaf provides photosynthetic assimilates to developing grains (Ghiglione *et al.*, 2008), the increased flag leaf length and width in the semi-dwarf lines is generally regarded as a positive pleiotropic effect. Moreover, spike length was either increased up to 16%, or was not significantly different from *Rht-B1b* or *Rht-B1a* (Table 1). Longer spikes increase the potential to carry more grains (Zamski, 1995), provided sufficient photo-assimilates are available.

**Table 2.**
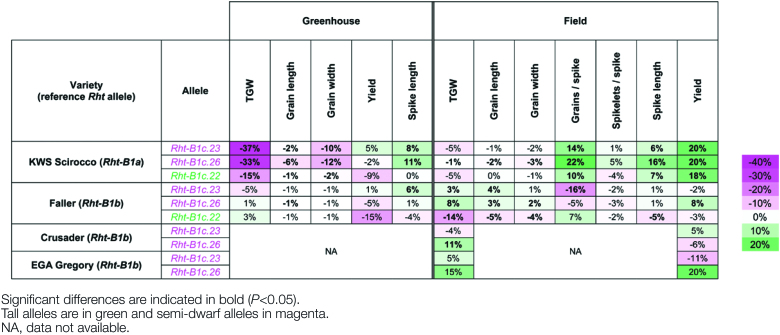
Effect of *ovg* alleles on grain size and yield in different wheat cultivars, in the greenhouse and field Percentage average difference of phenotypic traits for each *ovg* allele, relative to the reference allele, are shown. The scale next to the table details the intensity of color saturation for percentage difference. Greenhouse results were confirmed in at least two independent experiments. Representative data from the first experiment are shown.

Grain size, assessed via TGW, of greenhouse-grown *ovg* lines of Maringá was lower than that of *Rht-B1a*, yet higher than that of *Rht-B1c* and *Rht-B1b* (Fig. 3). Relative to *Rht-B1a*, in greenhouse-grown KWS Scirocco, TGW was reduced ~35% by *Rht-B1c.23* and *Rht-B1c.26*, and 15% by *Rht-B1c.22*, which corresponded to a proportionate reduction in both grain length and width (Fig. 1F; Table 2). While TGW only reduced ~5% in the field, the plot yield was higher because of a 10–22% increase in grain number per spike (Table 2). Interestingly, the number of spikelets per spike was not significantly affected, suggesting that this yield increase was caused by an increased grain number per spikelet (Table 2). Increased grain number has been previously described for *Rht-B1b* and *Rht-D1b*, and results from reduced floret abortion, a process during which developing florets enter a programmed cell death; hence it determines the final number of grains per spikelet (Youssefian *et al.*, 1992; Miralles *et al.*, 1998*b*). Although grain size was reduced relative to *Rht-B1a*, this negative effect was compensated by increased grain number, finally generating similar or larger yields than the wild type.

Relative to *Rht-B1b*, no negative *ovg* effects on TGW, grain length, grain width, or yield were observed in Faller, with the exception of field-grown *Rht-B1c.22* (Table 2). To assess further the *ovg* effect, relative to *Rht-B1b*, in different genetic backgrounds and environments, *Rht-B1c.23* and *Rht-B1c.26* were introgressed in two Australian spring wheat cultivars, Crusader and EGA Gregory, and grown in an Australian field trial. Both cultivars carry the *Rht-B1b* allele, hence the *ovg* alleles either slightly reduced (7%) or did not significantly affect plant height (Supplementary Fig. S2). Similar to Faller, no significant, negative effects on yield or TGW were detected (Table 2). In conclusion, the *ovg* alleles did not reduce overall grain or plot yield, suggesting that the *ovg* alleles could be used for wheat breeding.

### Effect of *ovg* alleles on pre-harvest sprouting

The exceptionally high dormancy of *Rht-B1c* has been suggested to be useful to increase PHS resistance (Flintham and Gale, 1982; Flintham *et al.*, 1997*b*). To test whether the *ovg* alleles retained the resistance to PHS, spikes were exposed to germination-inducing conditions. All KWS Scirocco *Rht-B1a* spikes strongly sprouted in a humid environment (Fig. 1H). Spikes of *Rht-B1c.23*, and especially *Rht-B1c.26*, sprouted less than *Rht-B1c.22* spikes (Fig. 1H). Similarly, *Rht-B1c.23* and *Rht-B1c.26* sprouted less than *Rht-B1b* in Faller (Fig. 1G), indicating that these alleles show improved sprouting resistance.

**Fig. 4.**
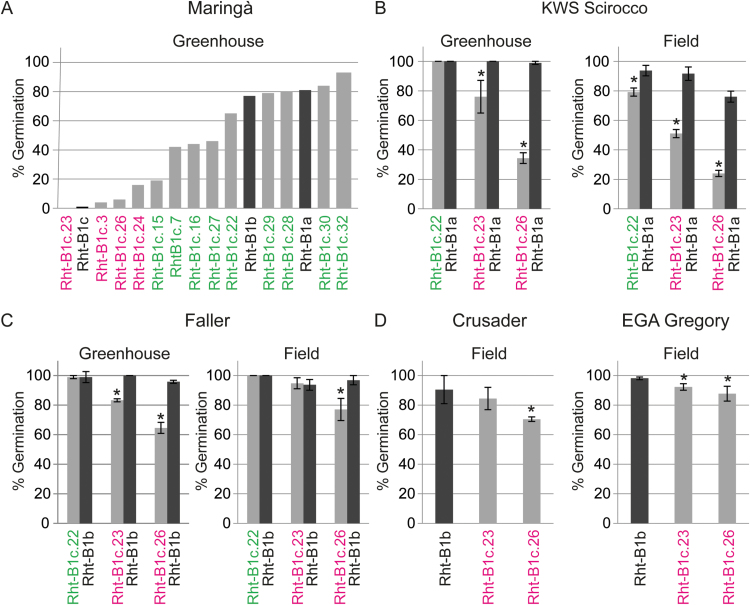
*ovg* alleles differentially affect dormancy in different cultivars in the greenhouse and field. Average germination ±SE at time points after harvest when the differences between *Rht-B1a* or *Rht-B1b* and the *ovg* alleles were at their maxima. The number of weeks of after-ripening varied between cultivar and environment, hence germination is shown for Maringá after 11 weeks (A); for KWS Scirocco after 10 weeks (greenhouse) and 3.5 weeks (field) (B); for Faller after 2 weeks (greenhouse) and 3.5 weeks (field) (C); for Crusader at harvest and for EGA Gregory after 1 week (D). * indicates a significant difference from *Rht-B1a* or *Rht-B1b* (*P*<0.05). Tall alleles are in green and semi-dwarf alleles are in magenta. For introgressed lines, results were confirmed in at least two sister line pairs, each derived from an independent BCF_1_ plant.

Since PHS is enhanced by a loss of dormancy, germination tests are currently regarded as the most accurate technique to quantify PHS resistance (Trethowan, 1995). We assessed germination of grains after harvesting each week, until germination reached at least 95% for all seed lots. Figure 4 shows germination at time points when the differences between *Rht-B1a* or *Rht-B1b* and *ovg* were at their maxima, yet all other time points showed a similar grouping of *ovg* alleles. In Maringá, the tall *ovg* alleles displayed a higher germination response than the semi-dwarf *ovg* alleles: when the differences between *Rht-B1a* and *ovg* were at their maxima, semi-dwarf alleles germinated <20%, while tall alleles, with the exception of *Rht-B1c.15*, germinated >40% (Fig. 4A). Although germination of the *ovg* semi-dwarfs was similar to the germination of *Rht-B1c* at this time point, *Rht-B1c* finally retained dormancy longer than the semi-dwarfing alleles (data not shown). In contrast to the effect of the *ovg* alleles on stem and coleoptile length, the dormancy of *Rht-B1b* was not intermediate between tall and semi-dwarf alleles, but rather similar to *Rht-B1a* (Fig. 4A). In KWS Scirocco, the differential *ovg* effect was confirmed in the greenhouse and field: *Rht-B1c.23* and *Rht-B1c.26* reduced germination by up to 65%, whereas *Rht-B1c.22* resulted in a maximal 10% reduction in germination, relative to *Rht-B1a* (Fig. 4B).

Although *Rht-B1a* and *Rht-B1b* result in comparable dormancy levels (Fig. 4A), the dormancy effect of the semi-dwarf alleles was lower in the three *Rht-B1b*-containing cultivars (i.e. germination never dropped below 60%) (Fig. 4C, D). However, dormancy is strongly influenced by genetic and environmental factors (Trethowan, 2001; Groos *et al.*, 2002; Mares and Mrva, 2014), which is demonstrated by the large differences in the time of after-ripening required for losing dormancy. The Australian cultivars lost dormancy more quickly than grains from the greenhouse or field trials in Germany: after 2 weeks, all Crusader and EGA Gregory grain samples showed >90% germination (data not shown). While Maringá, KWS Scirocco, and Faller have red grains, both Australian cultivars are white grained, which are generally less dormant (Mares and Mrva, 2014). However, besides these genetic factors, additional environmental factors cannot be excluded as causes for the rapid loss of dormancy. Although Faller and KWS Scirocco were grown in the same field conditions, the *ovg* effect on dormancy was lower in Faller than in KWS Scirocco. Since Faller has been developed for North America, this lower dormancy is probably a consequence of Faller having a different growth habit from KWS Scirocco (Figs 1A, B, 2), and being less adapted to the longer growing season in Europe. Furthermore, high variation in dormancy has also been shown between Arabidopsis ecotypes (Alonso-Blanco *et al.*, 2003).

Interestingly, the dormancy of *Rht-B1c.26* was twice as strong as that of *Rht-B1c.23* in KWS Scirocco (Fig. 4B), which was reproduced across all genetic backgrounds, albeit to a smaller extent (Fig. 4C, D). For instance, in the field trial with Faller, *Rht-B1c.23* lost dormancy completely, in contrast to *Rht-B1c.26* (Fig. 4C). In summary, *Rht-B1c.23*, and especially *Rht-B1c.26*, were more dormant than *Rht-B1c.22* in different environments and genetic backgrounds. These findings provide robust and reproducible evidence of the *ovg* effect on dormancy, and demonstrate the breeding potential of *Rht-B1c.23* and *Rht-B1c.26* for increasing PHS resistance.

### Effect of *ovg* alleles on falling number

**Fig. 5.**
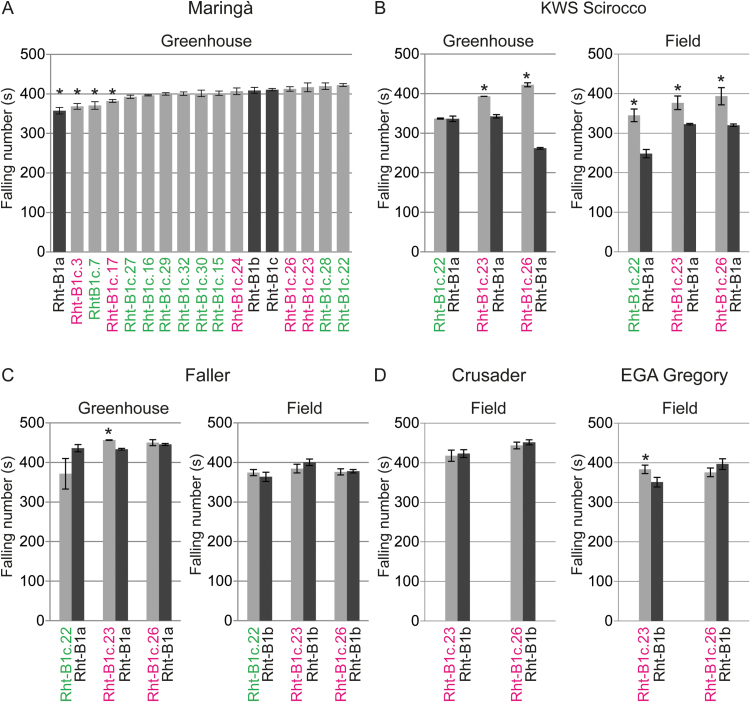
Effect of *ovg* alleles on falling number, independent of PHS, in different cultivars in the greenhouse and field. Average falling numbers ±SE are shown (*n*=3). * indicates a significant difference (*P*<0.05) from the reference allele, namely *Rht-B1c* for Maringá (A); *Rht-B1a* for KWS Scirocco (B); *Rht-B1b* for Faller (C), as well as for Crusader and EGA Gregory (D). Tall alleles are in green and semi-dwarf alleles are in magenta. For KWS Scirocco and Faller, results were confirmed in at least two sister line pairs, each derived from an independent BCF_1_ plant. For KWS Scirocco, results were additionally confirmed in a field trial in 2014 (Supplementary Fig. S4).

PHS strongly reduces falling number, which is a key parameter for quality assessment of grains. Besides PHS, falling number is determined by other genetic and environmental factors influencing the starch reserve of the endosperm, such as the production of α-amylase during late grain development in the absence of rain (Mares and Mrva, 2008, 2014). To verify whether the *ovg* alleles affect falling number, independent of PHS, the *ovg* effect on falling number was investigated when grain dormancy was completely lost, in conditions that did not trigger PHS (Fig. 5). Therefore, sprouting was not induced in the greenhouse, and field-grown material did not experience significant rainfall prior to harvest (Supplementary Fig. S3). In greenhouse-grown Maringá, all *ovg* alleles had a falling number up to 15% higher than that of *Rht-B1a*, while *Rht-B1b* and *Rht-B1c* had similar falling numbers (Fig. 5A). In addition, the falling number was well above 300 s, which is associated with excellent baking quality (Gooding *et al.*, 2012). Consequently, these data indicate that the *ovg* alleles have no negative effect on falling number.

In KWS Scirocco, some *Rht-B1a* sister lines had falling numbers <300 s. Interestingly, with the exception of greenhouse-grown *Rht-B1c.22*, all *ovg* alleles increased falling number in both greenhouse- and field-derived grains, with a maximum increase of 60% for *Rht-B1c.26* in the greenhouse (Fig. 5B). In general, falling number negatively correlates with α-amylase activity, as was demonstrated for field-grown KWS Scirocco grains (*R*^2^=0.7; Supplementary Fig. S5A). Consequently, a 20% increase in falling number by *Rht-B1c.23* or *Rht-B1c.26* was associated with a 37% reduction of α-amylase activity (Fig. 5B; Supplementary Fig. S5B). In contrast to KWS Scirocco, the effect of the *ovg* alleles in *Rht-B1b*-containing cultivars was limited (Fig. 5C, D). To summarize, the *ovg* alleles increased falling number compared with *Rht-B1a*, and correspond to that of the widely deployed semi-dwarf *Rht-B1b* allele, further strengthening their potential for wheat breeding.

## Discussion

### Novel second-site mutations in *Rht-B1c* show consistent GA-related effects across genetic backgrounds

Three *ovg* alleles (Rht-*B1c.22*, *Rht-B1c.23*, and *Rht-B1c.26*) exhibited strong and causal effects on vegetative organ growth, grain quality, and yield in different environments and genetic backgrounds (Figs 1–5; Table 1). First, these *ovg* alleles showed consistent effects in spring wheat cultivars with diverse growth characteristics. Secondly, the *ovg* effects were observed not only in independent greenhouse experiments, but also in Australian and German field trials, each representing very different climatic conditions. Thirdly, the backcrossed lines in four commercial spring wheat cultivars displayed phenotypes consistent with the original Maringá mutants, thereby ruling out that additional mutations might have caused the phenotype in the original mutated Maringá *Rht-B1c* lines.

### Phenotypic *ovg* effects reveal different regulatory mechanisms in GA responses across organs

**Fig. 6.**
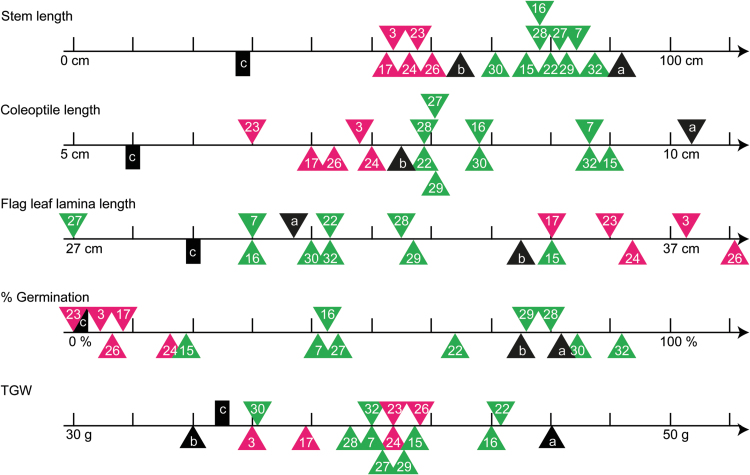
Summary of the phenotypic effect of the *ovg* alleles. Summary of data represented in Table 1 and Figs 3–5. Magenta and green triangles indicate the absolute average value for the semi-dwarf and tall *ovg* alleles, respectively. The number within the triangle indicates the *ovg* alleles, requiring the *Rht-B1c*.- prefix. Black triangles indicate the average phenotypic trait for *Rht-B1a* and *Rht-B1b*, designated with ‘a’ and ‘b’, respectively, whereas the black rectangle represents *Rht-B1c* (‘c’).

As summarized in Fig. 6, the second-site *ovg* mutations suppressed the dwarfism of *Rht-B1c* to different extents. This differential effect allowed classification of the 14 *ovg* alleles into nine slightly taller and five slightly shorter dwarfing alleles, relative to *Rht-B1b* (Fig. 6; Table 1). The grouping of *ovg* alleles into two groups that are shorter or taller than the standard semi-dwarfing *Rht-B1b* was similar for both coleoptile and peduncle length (Table 1). While similar grouping is still maintained when looking at the elongation of the first leaves, the *ovg* effect was weaker than for the other vegetative organs. For instance, in KWS Scirocco, *Rht-B1c.23* reduced stem and peduncle length by >30%, whereas the length of the first leaves was only reduced ~20% (Table 1). The lower heritability of leaf elongation, as compared with that of the peduncle (Supplementary Table S3), suggests that leaf elongation is more strongly controlled by environmental factors, thereby buffering the phenotypic impact of the *ovg* alleles.

In contrast to the *ovg* effects on first leaves and internodes, the *ovg* alleles had an inverse effect on flag leaf lamina length: semi-dwarf alleles, which mildly restored the effect of *Rht-B1c* on stem length, had the longest flag leaves (Fig. 6; Table 1), highlighting that different DELLA-regulating mechanisms are important in flag leaf elongation as compared with stem elongation. The deviating morphology of the flag leaf compared with the other leaves has been reported previously (Rawson *et al.*, 1983; McCaig and Morgan, 1993; Calderini *et al.*, 1996), and is probably linked to its role in providing photosynthetic assimilates to the developing grains (Ghiglione *et al.*, 2008). These findings illustrate that the DELLA-driven elongation responses most probably depend on their interaction with development- and organ-specific factors. Since DELLA has been shown to function as an integrator of environmental and endogenous cues through direct interaction with a plethora of regulatory proteins, it is likely that different organs contain different sets of DELLA-interacting proteins (Davière and Achard, 2013; Locascio *et al.*, 2013).

All *ovg* alleles showed a higher TGW than either *Rht-B1b* or *Rht-B1c* (Figs 3, 6). The smaller grain, hence lower TGW, of *Rht-B1b* is a known drawback of this allele. Miralles *et al.* (1998*a*) demonstrated that *Rht-B1b* and *Rht-D1b* reduced grain size compared with *Rht-B1a* by reducing cell division, whereas internodes were shortened by inhibiting cell elongation. Consequently, the mechanism by which different DELLA mutations regulate grain size needs further elucidation.

### Dormancy and elongation are coupled in *Rht-B1c* and *ovg* mutants, but not in Green Revolution mutants

Due to the widespread use of *Rht-B1b* for reducing plant height, its pleiotropic effects relative to *Rht-B1a* have been characterized in detail over the past decades, and were confirmed in this study. Without changing the timing of developmental events (Fig. 2; Youssefian *et al.*, 1992*b*), *Rht-B1b* reduces the length of vegetative organs down to 20–25% (Table 1; Fig. 6; Flintham *et al.*, 1997*b*; Rebetzke *et al.*, 2007), with the exception of the flag leaf (Table 1; Fig. 6; McCaig and Morgan, 1993; Calderini *et al.*, 1996). *Rht-B1b* further leads to a 20% decrease of TGW (Figs 3, 6; Miralles and Slafer, 1996), while increasing grain number (Table 2; Youssefian *et al.*, 1992; Miralles and Slafer, 1996; Miralles *et al.*, 1998*b*). Remarkably, *Rht-B1b* does not affect dormancy; hence it germinates as readily as *Rht-B1a* (Figs 4, 6; Gooding *et al.*, 2012). Similarly, increased expression of *Rht-D1b* in the *Rht-D1c* mutant also further increased dwarfism without affecting dormancy (Gooding *et al.*, 2012). Although both elongation and dormancy are typical DELLA-regulated GA responses (reviewed by Graeber *et al.*, 2012; Martínez *et al.*, 2016), these findings suggest that the Green Revolution alleles affect elongation without affecting dormancy; in other words, *Rht-B1b* and *Rht-D1b* uncouple the elongation and dormancy responses.

**Fig. 7.**
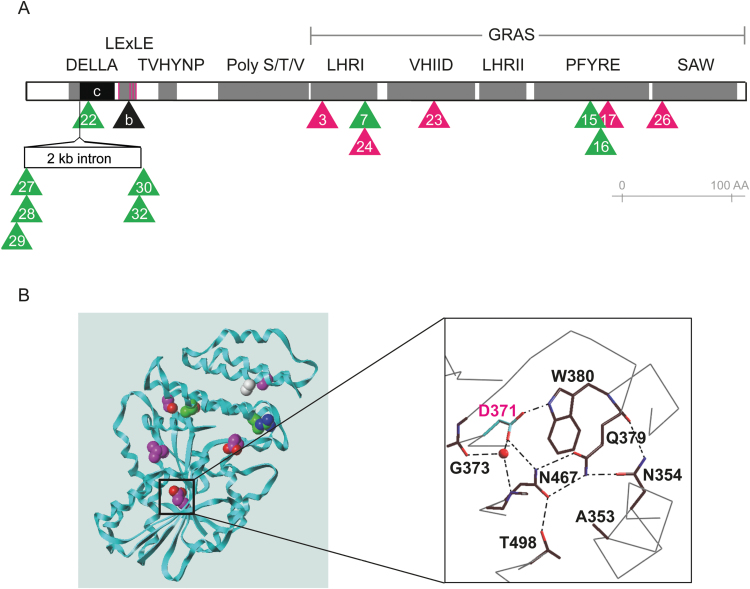
The position of the *ovg* second-site mutations on the DELLA protein. (A) Position of the *ovg* second-site mutations over the different motifs and domains of the DELLA protein. The black box indicates the predicted 30 amino acid *Rht-B1c* insertion, whereas the black triangle shows the position of the *Rht-B1b* stop codon. Magenta lines in the LExLE motif represent positions of putative translational reinitiation. Magenta and green triangles indicate the position of the semi-dwarf and tall *ovg* alleles, respectively. The number within the triangle indicates the *ovg* alleles, requiring the Rht-B1c.- prefix. The scale bare shows the length of 100 amino acids. (B) Positions of the amino acids altered by the C-terminal *ovg* mutations on the crystal structure of the OsSCL7 GRAS protein (Li *et al.*, 2016). Carbon atoms of semi-dwarf and tall *ovg* alleles are shown in magenta and green, respectively. Gray carbons represent the position of *Rht-B1c*.7 and *Rht-B1c.24*. Nitrogen and oxygen atoms are colored in blue and red, respectively. The inset shows the amino acids surrounding the *Rht-B1c.23* (D371) position, where dashed lines represent hydrogen bonds and the red dot represents a water molecule.

In contrast to *Rht-B1b* and *Rht-D1b*, the extreme dwarfism of *Rht-B1c* is associated with strong dormancy (Flintham and Gale, 1982; Flintham *et al.*, 1997*a*; Gooding *et al.*, 2012). Furthermore, the results presented here show that this association persists when *Rht-B1c* partially loses its suppressive function: semi-dwarf *ovg* lines are more dormant than tall *ovg* lines (Figs 4, 6). Consequently, dormancy and elongation are coupled in *Rht-B1c* and its *ovg* derivatives, yet uncoupled in *Rht-B1b* and *Rht-D1b*, thereby highlighting a remarkable difference between *Rht-B1c* and the widely deployed Green Revolution alleles. Although no biochemical evidence is available, DNA sequencing suggests that the DELLA proteins produced by *Rht-B1b* or *Rht-D1b*, and *Rht-B1c* strongly differ. Whereas RHT-B1C contains a 30 amino acid insertion following the DELLA motif, *Rht-B1b* and *Rht-D1b* encode a nucleotide substitution that introduces a premature stop codon in the LExLE motif. It has been suggested that translational reinitiation, at one of the three AUG start codons that are immediately downstream of this stop codon (Fig. 7A), could lead to the production of N-terminally truncated proteins, lacking ~10% (66–70 amino acids) of the total protein (Peng *et al.*, 1999; Pearce *et al.*, 2011). Since these truncated DELLA proteins lack the DELLA motif, they are insensitive to the GA-induced degradation mechanism and presumably accumulate (Peng *et al.*, 1999; Pearce *et al.*, 2011), thereby repressing elongation without affecting dormancy. Consequently, the first 70 amino acids of the DELLA protein have a unique role in regulating dormancy, but not in the control of elongation.

The first 70 amino acids of the DELLA protein have recently been shown to be important for the transactivation activity and post-translational modifications of the DELLA protein. For example, the deletion of the DELLA motif reduced the transactivation activity of the DELLA protein in rice (Hirano *et al.*, 2010). In Arabidopsis, glycosylation also reduced the suppressive function of DELLA proteins, and occurred preferentially in the N-terminal half of the protein (Zentella *et al.*, 2016). Interestingly, several transcriptomic approaches demonstrated that there is only a marginal overlap of DELLA-regulated genes between different organs (Locascio *et al.*, 2013). Therefore, the DELLA protein probably regulates a different set of genes in elongation from that in dormancy. Consequently, we hypothesize that the first 70 amino acids of the DELLA protein are involved in regulating gene expression in dormancy, but not in elongation.

### Application potential: improving pre-harvest sprouting resistance

Although the high dormancy of *Rht-B1c* is beneficial for PHS resistance, its extreme dwarfism prevented its use in commercial cultivars (Flintham and Gale, 1982; Flintham *et al.*, 1997*a*). In this respect, this study reveals a unique application potential: *Rht-B1c.23* and *Rht-B1c.26* were more dormant than *Rht-B1a* or *Rht-B1b*, in different genetic backgrounds and environments, which correlated with improved PHS resistance (Figs 1G, H, 4). In addition, final height was within the range of standard semi-dwarfing alleles, whereas no negative effects on overall yield or falling number were observed (Table 2; Fig. 5). Consequently, *Rht-B1c.23* and *Rht-B1c.26* could be used to adjust plant height while improving PHS resistance in regions with frequent rainfall prior to harvest.

### *Ovg* mutations increase insight into DELLA protein function

Together, the described phenotypes emphasize that the amino acid residues mutated in the *ovg* alleles are crucial for different GA responses in wheat. Figure 7A depicts the position of the second-site *ovg* mutations on the RHT-B1C protein. At the N-terminal half, which consists of the DELLA, LExLE, TVHYNP, and poly S/T/V motifs, five *ovg* alleles probably result in a lower splicing efficiency of the *Della* transcript and, in turn, in less DELLA protein (Fig. 7A; Supplementary Table S1). The sixth N-terminal *ovg* allele, *Rht-B1c.22*, contains a stop codon within the non-spliced *Rht-B1c* insertion (Fig. 7A; Supplementary Table S1). In analogy with *Rht-B1b* and *Rht-D1b*, translational reinitiation could occur at one of the four following AUG start codons, thereby generating a low level of N-terminally truncated DELLA proteins. Taken together, the N-terminal *ovg* mutations probably reduce DELLA protein levels, which results in the tall *ovg* phenotype. Although validation of this hypothesis at the protein level is required, the absence of a suitable assay for detecting DELLA protein in wheat renders this experiment at least problematic (Pearce *et al.*, 2011).

The other amino acid substitutions occurred within strongly conserved residues of the C-terminal GRAS domain of the DELLA protein (Fig. 7A; Chandler and Harding, 2013). This GRAS domain characterizes a plant-specific family of transcriptional regulators and contains five conserved subdomains: leucine heptad repeat I (LHR I), VHIID, leucine heptad repeat II (LHR II), PFYRE, and SAW (Pysh *et al.*, 1999). Interestingly, some of those mutations were highlighted as important residues for DELLA function in other plant species. Substitutions identical to *Rht-B1c.15* and *Rht-B1c.24* in the PFYRE and LHRI subdomain, respectively, were identified in a suppressor screen in the GA-insensitive *Sln1d* barley mutant (Chandler and Harding, 2013). Alanine scanning in rice plants that overexpressed DELLA revealed that the residue mutated in the *Rht-B1c.15* line is required for growth repression (Hirano *et al.*, 2010). A suppressor screen in the Arabidopsis GA biosynthesis mutant *ga1-3* identified a substitution of the amino acid adjacent to the residue mutated in the *Rht-B1c.17* line, named *rga-2* (Silverstone *et al.*, 1998). Together, the amino acid residues substituted in the *ovg* alleles appear crucial for the DELLA-regulated growth mechanism in different plant species.

The recent characterization of the crystal structure of the rice SCARECROW-LIKE 7 (OsSCL7) GRAS protein further aids our understanding of the importance of specific amino acid residues for DELLA function (Li *et al.*, 2016). Mapping the amino acid mutations of the *ovg* alleles on OsSCL7, based on homology between the GRAS domain of RHT-B1 and OsSCL7, reveals that the C-terminal *ovg* mutations are not clustered together, but presumably are located at different positions of the protein (Fig. 7B). Although the majority of mutated amino acids are positioned at the interior of the protein, RHT-B1C.26 (E579K) is positioned at the protein surface. Therefore, the *Rht-B1c.26 ovg* substitution from glutamic acid to lysine, which alters the charge at this position from negative to positive, could directly affect interactions with other proteins. In addition, in OsSCL7, this glutamic acid residue is in close proximity to some residues of the PFYRE subdomain, potentially forming a binding pocket (Supplementary Fig. S6). However, this PFYRE region shows limited conservation in *Rht-B1*. In contrast, the aspartic acid at the *Rht-B1c.23* position (D371) forms hydrogen bonds with three residues of the VHIID (G373 and W380) and PFYRE (N467) subdomain (Fig. 7B), which are conserved across monocot species. In turn, these residues form hydrogen bonds with other conserved residues of the VHIID (A353, N354, Q379) and PFYRE (T498) subdomain. The *Rht-B1c.23 ovg* substitution from aspartic acid to asparagine is likely to disrupt some of these hydrogen bonds through conformational changes. Interestingly, the importance of these hydrogen bonds for the functionality of the DELLA protein is supported by the observation that substitution of QWP379-381 by three alanine residues results in a loss-of-function phenotype in rice (Hirano *et al.*, 2010). Furthermore, this alanine substitution reduced the interaction between the DELLA protein and GID1 in a yeast two-hybrid assay, suggesting that conformational changes of DELLA can reduce the affinity for specific interaction partners (Hirano *et al.*, 2010). These findings further support the hypothesis that the binding affinity for specific proteins is differentially affected by semi-dwarf and tall *ovg* alleles. Alternatively, these conformational changes could increase the degradation rate of the DELLA protein.

In summary, the *ovg* amino acid substitutions partially released the growth repression caused by the N-terminal *Rht-B1c* insertion. Their functional importance in different GA responses and species highlights the value of the *ovg* alleles in elucidating DELLA function in different organs and growth responses. Furthermore, the coupling of dormancy and elongation in *Rht-B1c* and its *ovg* derivatives can shed light on the importance of the first 70 amino acids of the DELLA protein, and can now be agronomically exploited via *Rht-B1c.23* and *Rht-B1c.26*.

## Supplementary data

Supplementary data are available at *JXB* online.

Table S1. Nucleotide and amino acid substitutions characteristic for each *ovg* allele.

Table S2. Phenotypic measurements on main stem correlated with measurements on the first and second tiller in Maringá.

Table S3. Reproducibility of *ovg* effects in KWS Scirocco.

Fig. S1. *Rht-B1c.26* reduced the final length of each internode in Maringá.

Fig. S2. Effect of *Rht-B1c.23* and *Rht-B1c.26* on plant height in Crusader and EGA Gregory.

Fig. S3. Rainfall in Gatersleben (Germany) and Yanco (New South Wales, Australia).

Fig. S4. *ovg* alleles increased falling number in KWS Scirocco field trial.

Fig. S5. Negative correlation between falling number and α-amylase activity.

Fig. S6. Position of *Rht-B1c.26* on the crystal structure of the OsSCL7 GRAS protein (Li *et al.*, 2016).

## Supplementary Material

supplementary_tables_S1_S3_figures_S1_S6

## Abbreviations:

BC: backcross
GA: gibberellin
KASP: Kompetitive Allele Specific PCR
ovg: overgrowth
PHS: pre-harvest sprouting
SRC: soil retention capacity
TGW: thousand grain weight.
