# Supplementary material for: Differential coupling of gibberellin responses by Rht-B1c suppressor alleles and Rht-B1b in wheat highlights a unique role for the DELLA N-terminus in dormancy
Source: J Exp Bot. 2017 Jan 9;68(3):443–55. doi: 10.1093/jxb/erw471 (PMC5853533; doi:10.1093/jxb/erw471)
Supplement: supplementary_tables_S1_S3_figures_S1_S6 [file erw471_suppl_supplementary_tables_s1_s3_figures_s1_s6.pdf]

Differential coupling of GA responses by Rht-B1c suppressor alleles and Rht-B1b in wheat highlight a unique role of the DELLA N-terminal in dormancy

Karel Van De Velde[1,3], Peter Chandler[2], Dominique Van Der Straeten[3], Antje Rohde[1]

[1] R&D Innovation Center, Bayer CropScience, Ghent, Belgium

[2] CSIRO Plant Industry, Canberra ACT 2601, Australia

[3] Ghent University, Department of Physiology, Laboratory of Functional Plant Biology, Ghent, Belgium

Contact: antje.rohde@bayer.com

Supplemental tables

Table S1: Nucleotide and amino acid substitutions characteristic for each *ovg* allele.

Shown are the positions of changed nucleotides or AAs of the *Rht-B1c* sequence (accession number JF930279), as characterized by Chandler and Harding (2013, 2014). '*' indicates premature termination. 'Splice' indicates that the mutations involve the two nucleotides at the acceptor or the donor splice site on either side of the 2kb insertion.

**Allele**	Nucleotide	Amino acid
Rht-B1c.3	G2747A	A271T
Rht-B1c.7	C2865T	A310V
Rht-B1c.15	G3477A	R514H
Rht-B1c.16	C3507T	T524I
Rht-B1c.17	C3519T	S528F
Rht-B1c.22	CC2108-2109TA	P58*
Rht-B1c.23	G3047A	D371N
Rht-B1c.24	G2864A	A310T
Rht-B1c.26	G3671A	E579K
Rht-B1c.27	G148A	splice
Rht-B1c.28	G148T	splice
Rht-B1c.29	G147A	splice
Rht-B1c.30	G2084A	splice
Rht-B1c.32	G2083A	splice

Table S2: Phenotypic measurements on main stem correlated with measurements on first and second tiller in Maringá.

For different phenotypic traits, Pearson's correlation coefficient between main stem and first or second tiller are shown (n = 160). Traits measured in Maringá for 14 *ovg* alleles, *Rht-B1a* and *Rht-B1b*.

	**Tiller 1**	**Tiller 2**
**Stem**	0.83	0.82
**Peduncle**	0.70	0.68
**Penultimate internode**	0.72	0.66
**Flag leaf lamina**	0.45	0.15
**Flag leaf sheath**	0.55	0.45
**Spike**	0.64	0.66

Table S3: Reproducibility of *ovg* effects in KWS Scirocco.

Broad-sense heritability and percentage average difference of phenotypic traits for *Rht-B1c.23* and *Rht-B1c.26*, relative to Rht-B1a, are shown. The scale below the table details the intensity of color saturation for percent difference. Significant differences are indicated in bold (p<0.05).

BC2F3 KWS Scirocco plants were grown in two independent greenhouse experiments (GH1 and GH2) and in the field. In experiment GH1, all plants were well-watered (60% SRC, control), while in experiment GH2, plants were grown under either well-watered (60% SRC, control) or constant drought conditions (30% SRC). Field plots of 1.5 m by 2.5 m were sown in Gatersleben (Germany) on 14 April 2014 in a randomized block design consisting of three replicated plots for each sister line.

	Rht-B1c.23				Rht-B1c.26				Heritabiliy
	GH1	GH2	GH2 drought	Field	GH1	GH2	GH2 Drought	Field	
**Main stem length**	**-35%**	**-31%**	**-25%**	**-20%**	**-35%**	**-30%**	**-23%**	**-24%**	0.81
**Peduncle length**	**-23%**	**-23%**	**-22%**	**-23%**	**-30%**	**-26%**	**-24%**	**-22%**	0.67
**Flag leaf sheath length**	**-16%**	**-8%**	**-10%**	-2%	**-13%**	**-7%**	**-8%**	-4%	0.68
**Flag leaf lamina length**	1%	**11%**	**-11%**	**-7%**	-5%	5%	**-16%**	-2%	0.34

**Scale:**	-40%	-20%	0	20%

Supplemental figures

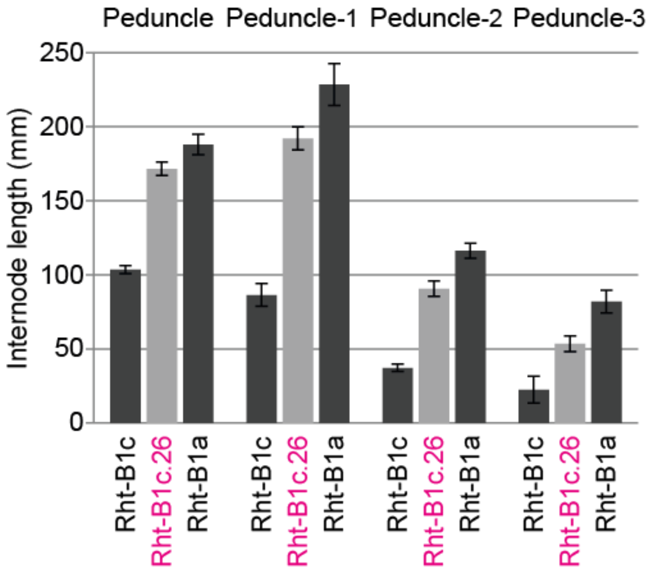

Figure S1: *Rht-B1c.26* reduced the final length of each internode in Maringá.

Average internode length ± SE of the main stem, measured on eight Maringá plants per allele, are shown. Peduncle and subsequent lower internodes were designated peduncle, peduncle-1, peduncle-2, peduncle-3.

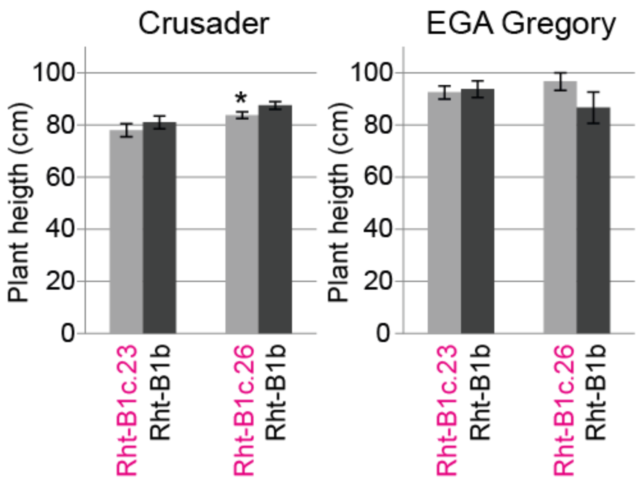

Figure S2: Effect of *Rht-B1c.23* and *Rht-B1c.26* on plant height in Crusader and EGA Gregory.
* indicates significant difference to *Rht-B1b* ($p<0.05$), determined via paired t-test on three to five sister line pairs, for each allele and cultivar.

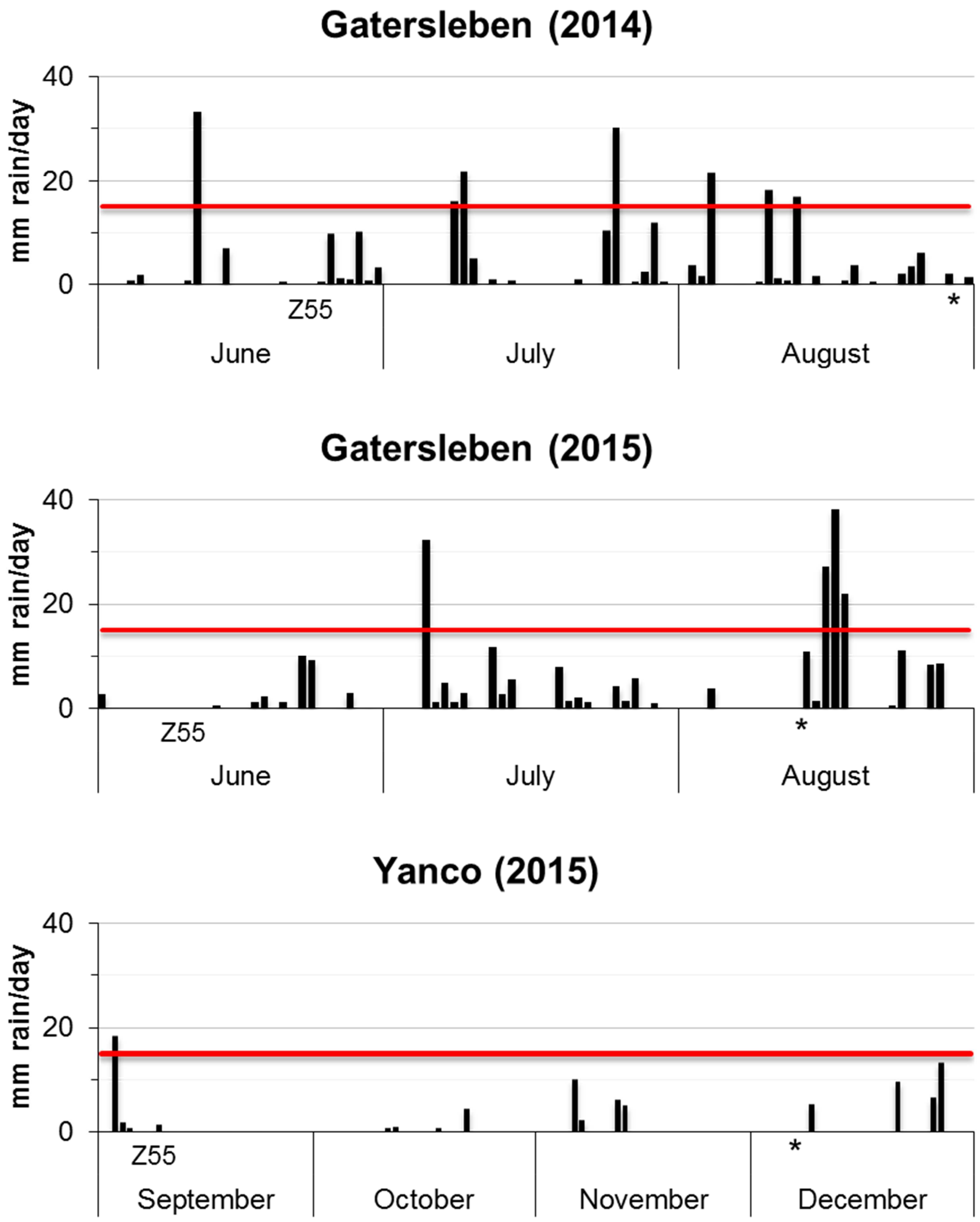

Figure S3: Rainfall in Gatersleben (Germany) and Yanco (New South Wales, Australia).

Daily rainfall between plant heading and harvesting is shown. Red line indicates average minimal rainfall required during two subsequent days to induce preharvest sprouting in harvest-ripe wheat plants (Mares and Mrva, 2014). * indicates harvesting date; Z55 indicates Zadok growth stage 55 (heading).

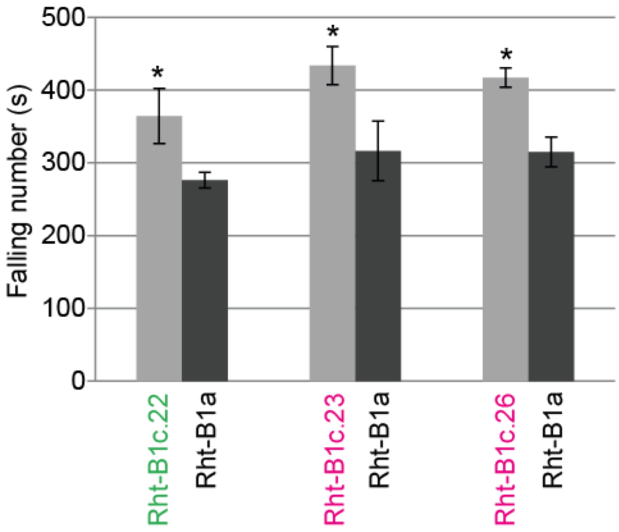

Figure S4: *ovg* alleles increased falling number in KWS Scirocco field trial.

Average falling numbers ±SE of field-harvested BC2F4 KWS Scirocco grains. Field plots of 1.5 m by 2.5 m were sown in Gatersleben (Germany) on 14 April 2014 in a randomized block design consisting of three replicated plots for each sister line. * indicates significant difference to *Rht-B1a* ($p<0.05$). Tall *ovg* alleles in green, semi-dwarf *ovg* alleles in magenta.

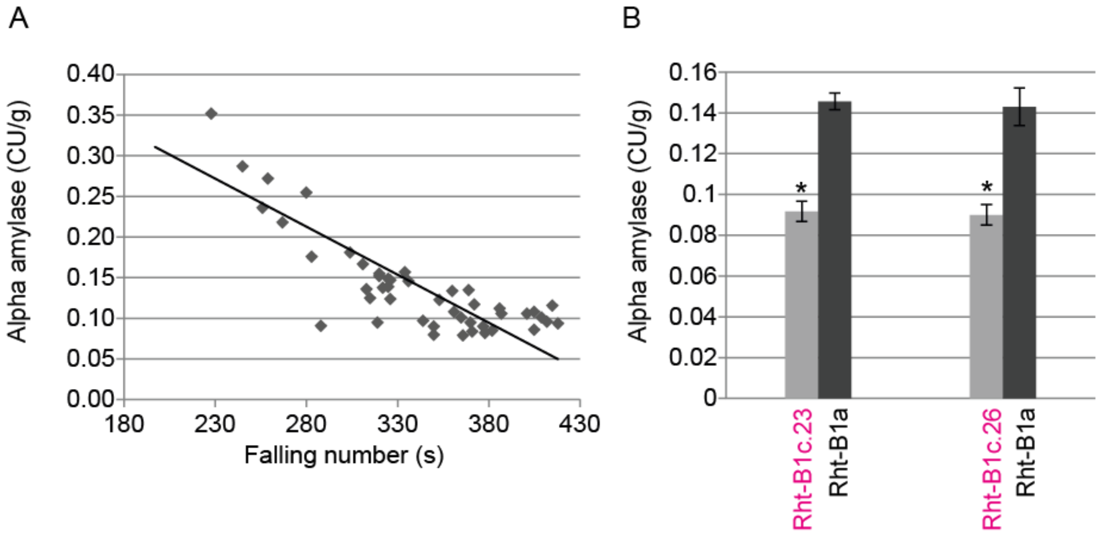

Figure S5: Negative correlation between falling number and α-amylase activity.

KWS Scirocco (BC3F4) grain samples of 350g per plot were milled. Subsequently, 7 g flour was used for falling number and 1 g flour for α-amylase activity, according to the international standard methods ICC-No. 107/1 and ICC-No. 303, respectively. The latter was executed using the Ceralpha method (Megazyme®, County Wicklow, Ireland, cat no. K-CERA) with blocked p-nitrophenol maltoheptaoside (BPNPG7), and expressed as Ceralpha units (CU), which correspond to the amount of enzyme required to release one micromole of p-nitrophenol from BPNPG7 in one minute.

A) Scatter plot showing correlation between alpha-amylase activity (Ceralpha units/gram grain) and falling number (seconds) ($R^2 = 0.70$).

B) Average alpha-amylase activity (Ceralpha units/gram grain) ±SE are shown. * indicates significant difference to *Rht-B1a* sister line ($p<0.05$). Results were confirmed in at least three sister line pairs, each derived from an independent BCF1 plant. Semi-dwarf *ovg* alleles in magenta.

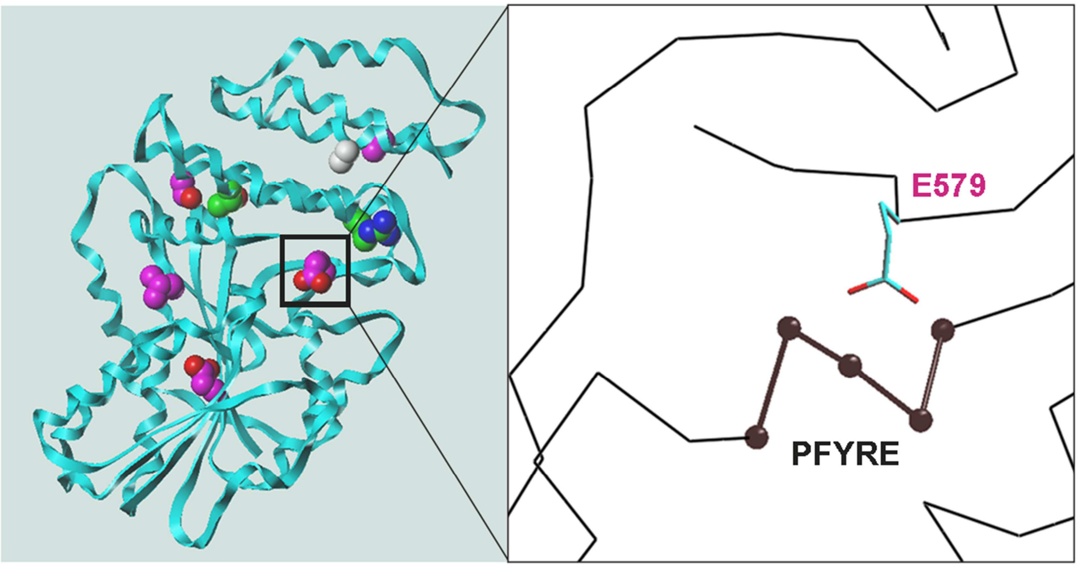

Figure S6: Position of *Rht-B1c.26* on the crystal structure of the OsSCL7 GRAS protein (Li *et al.*, 2016).

Carbon atoms of semi-dwarf and tall *ovg* alleles shown in magenta and green, respectively. Grey carbons represent position of *Rht-B1c*.7 and *Rht-B1c.24*. Nitrogen and oxygen atoms are colored in blue and red, respectively. Inset shows the amino acids of the PFYRE subdomain surrounding the *Rht-B1c.26* (E579) position.
